# Frugivory and Spatial Patterns of Seed Deposition by Carnivorous Mammals in Anthropogenic Landscapes: A Multi-Scale Approach

**DOI:** 10.1371/journal.pone.0014569

**Published:** 2011-01-21

**Authors:** José V. López-Bao, Juan P. González-Varo

**Affiliations:** 1 Department of Conservation Biology, Estación Biológica de Doñana (CSIC), Seville, Spain; 2 Departamento de Biología Vegetal y Ecología, Universidad de Sevilla, Seville, Spain; Stanford University, United States of America

## Abstract

**Background:**

Knowledge about how frugivory and seed deposition are spatially distributed is valuable to understand the role of dispersers on the structure and dynamics of plant populations. This may be particularly important within anthropogenic areas, where either the patchy distribution of wild plants or the presence of cultivated fleshy-fruits may influence plant-disperser interactions.

**Methodology/Principal Findings:**

We investigated frugivory and spatial patterns of seed deposition by carnivorous mammals in anthropogenic landscapes considering two spatial scales: ‘landscape’ (∼10 km^2^) and ‘habitat type’ (∼1–2 km^2^). We sampled carnivore faeces and plant abundance at three contrasting habitats (chestnut woods, mosaics and scrublands), each replicated within three different landscapes. Sixty-five percent of faeces collected (n = 1077) contained seeds, among which wild and cultivated seeds appeared in similar proportions (58% and 53%) despite that cultivated fruiting plants were much less abundant. Seed deposition was spatially structured among both spatial scales being different between fruit types. Whereas the most important source of spatial variation in deposition of wild seeds was the landscape scale, it was the habitat scale for cultivated seeds. At the habitat scale, seeds of wild species were mostly deposited within mosaics while seeds of cultivated species were within chestnut woods and scrublands. Spatial concordance between seed deposition and plant abundance was found only for wild species.

**Conclusions/Significance:**

Spatial patterns of seed deposition by carnivores differed between fruit types and seemed to be modulated by the fleshy-fruited plant assemblages and the behaviour of dispersers. Our results suggest that a strong preference for cultivated fruits by carnivores may influence their spatial foraging behaviour and lower their dispersal services to wild species. However, the high amount of seeds removed within and between habitats suggests that carnivores must play an important role – often overlooked – as ‘restorers’ and ‘habitat shapers’ in anthropogenic areas.

## Introduction

Ecological processes associated with frugivory, seed dispersal and recruitment of endozoochorous plants are spatially structured due not only to plant distribution and habitat heterogeneity [1], [2], but also to the local abundance and behaviour of seed dispersers [3], [4]. The spatial scale at which plant-disperser interactions occur may determine the distribution, dynamics and genetic structure of plant populations and, therefore, of plant species assemblages [5]–[7].

Despite the well-known role of carnivorous mammals (Carnivora) as fruit consumers and seed dispersers [8]–[10], during the past three decades birds have captured almost all of the attention devoted to the study of frugivory and seed dispersal in temperate climate zones [11], [12]. These studies have addressed a wide range of topics, including spatio-temporal variations in frugivore assemblages and frugivory, seed rain patterns, as well as their implications for the demography of plant populations [11]–[14]. However, most studies performed to date with carnivores as legitimate seed dispersers have focused on a mere description of mutualistic relationships [8], [15]–[17] or on the evaluation of the effects of gut passage on seed viability and germination [18]–[20], and only recently new topics have been addressed [21], [22].

During the last decade, the spatial scale at which plant-disperser interactions are distributed have been addressed on different plant-frugivore systems, although mostly including birds [1], [2], [23], [24]. However, none of the previous studies dealing with carnivores as seed dispersers have included or analyzed the mutualistic interaction at more than one spatial scale [8], [15], [19]–[21], [25]. Therefore, the spatial scale at which the ecological processes involving carnivores and fleshy-fruited plants take place remains largely unexplored [25], [26]. Carnivores greatly differ from birds in terms of feeding behaviour, mobility, gut retention time, habitat use and spatial patterns of seed deposition [20], [25], [27], which are pivotal features in determining the spatial scale of plant-disperser interactions. For instance, differences in spatial mobility between frugivores may determine whether the patterns of frugivory and seed deposition are mostly influenced by local patch features or landscape configuration, which has important ecological implications in the dispersal ecology of plants [23]. This information is essential to understand the role of carnivores as dispersal vectors in a spatial context.

Anthropogenic landscapes are characterized by the transformation of the original vegetation cover into ‘man-made’ habitats such as managed forests, agricultural fields, orchards and pastures [28]. Hence, these landscapes typically are comprised of habitat patches that differ greatly in vegetation structure and composition, degree of perturbation, successional stage and current management [29]. Such spatial heterogeneity determines non-uniform distribution of fleshy-fruited species, which is expected to shape spatial patterns of carnivore-mediated seed deposition. Furthermore, anthropogenic systems may provide resources to fruit consumers in the form of cultivated fleshy-fruits [30], which may interfere with the dispersal mutualism of wild plant species [15]. Thus, the importance of studies of carnivore-mediated seed deposition in anthropogenic landscapes at multiple spatial scales is two-fold: (i) to understand the influence of the patchy distribution of plants on this mutualism, and (ii) to gauge the influence of cultivated fruits on native plant-carnivore interactions. Emergent information will improve our knowledge about the functioning of human-modified ecosystems in terms of plant-carnivore interactions [31] and, ultimately, about the services that carnivores provide as ‘restorers’ of fleshy-fruited plant assemblages within anthropogenic areas [25].

We investigated frugivory and the spatial patterns of seed deposition by carnivorous mammals in O Courel Mountains (NW Spain), where contrasting habitat types can be found as a result of the long-standing process of traditional human management [32], [33]. We considered two spatial scales: landscapes within the region and habitat types within the landscapes, in order to evaluate at which scale carnivore-mediated seed deposition is mostly structured.

Specifically we addressed three main questions: (1) how important are wild and cultivated fleshy-fruits in the diet of carnivorous mammals within anthropogenic landscapes?; (2) are seed deposition patterns structured at broad (landscape) and/or narrow (habitat) spatial scales considering different fruit types (all fleshy-fruited species together, wild and cultivated fleshy-fruited species separately and individual fleshy-fruited species)?; and (3) is the quantity of seed deposition associated with local plant abundance? We predict contrasting patterns of deposition of wild and cultivated seeds given that both types of fruits differ considerably (1) in nutritive reward (higher in cultivated fruits because they are larger in size and have a lower seed-burden than wild fruits) [15], [34], thus, on fruit preferences by carnivores; and (2) in spatial distribution across scales because the distribution of cultivated fruits depends on agricultural habitats, fully extended in all landscapes, whereas the distribution of wild fruits depends on species-specific favourable habitats.

## Methods

### Study area and fleshy-fruited species

**Figure 1:**
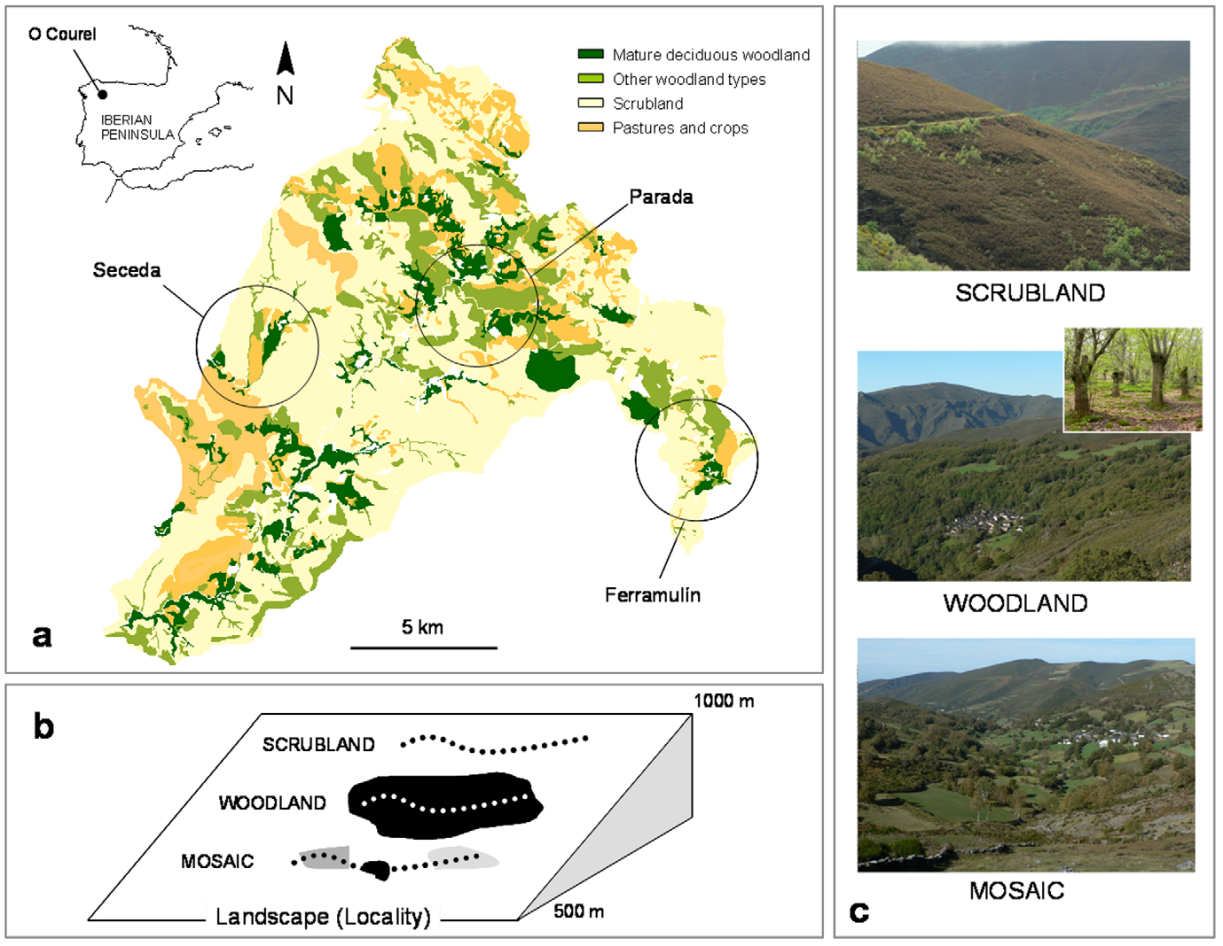
Study area and sampling design. A) Geographical location of the O Courel Mountains (NW Spain) showing the location of the three landscapes (valleys) included in this study (Seceda, Parada and Ferramulín). B) Sampling design of the study. C) Pictures of the three habitat types considered in this study. The figure shows the general spatial organization of the three different habitat types within each landscape. Broken lines represent the different transects made for sampling both carnivore faeces and plant abundance.

The study was conducted in the O Courel Mountains (NW Spain), a montane area of ca. 25000 hectares with elevation ranges between 450–1600 m. a.s.l. (Fig. 1). This region has traditionally been managed and severely modified by human activities, mainly livestock and agriculture. Consequently, successional scrublands (composed by *Erica australis*, *E. arborea* and *Calluna vulgaris*) and agricultural fields occupy most of the territory (Fig. 1A, 1C). One-third of the area is currently covered by woodlands, mainly sweet chestnut (*Castanea sativa*) woods and deciduous woodlands dominated by Pyrenean oaks (*Quercus pyrenaica*). Chestnut woods are traditional plantations of large trees (∼1m trunk diameter, many of them over 200 years old) surrounding small villages (<100 inhabitants), and managed for chestnuts and timber.

The most abundant wild fleshy-fruited species occurring in the area are bramble (*Rubus* spp.), hawthorn (*Crataegus monogyna*), alder buckthorn (*Frangula alnus*), rowan (*Sorbus aucuparia*), blackthorn (*Prunus spinosa*) and rose (*Rosa* spp.). Moreover, cultivated species are very common within the villages and the surrounding area, both in agricultural fields and within chestnut woodlands; the most common cultivated fleshy-fruited species are common fig (*Ficus carica*), cherry tree (*Prunus avium*), apple tree (*Malus domestica*), plum tree (*Prunus domestica*) and pear tree (*Pyrus communis*). Whereas recruitment is regular in wild species, germination and establishment of cultivated fleshy-fruited species is rare in semi-natural habitats except for *Prunus avium* (unpublished data) [19]. On the other hand, the dramatic process of depopulation and land abandonment occurred in O Courel (as in many other rural areas of Europe) during the last decades have markedly reduced the amount of cultivated fruits harvested by people. It is worth mentioning that between 1970 and 2000 human population decrease by 60%, farms by 40% and cattle by 80%; whereas at the habitat scale, the cover percentage of woodlands increase by 35% in detriment of crops and scrublands, which undergo a reduction of 13% and 20%, respectively [32]. As a consequence, the availability of cultivated fruits to wildlife has increased in the last years.

Carnivorous mammals are well represented in O Courel Mountains [35]. There are several frugivores and therefore potential seed dispersers in the area: red fox (*Vulpes vulpes*), stone marten (*Martes foina*), pine marten (*Martes martes*), Eurasian badger (*Meles meles*), common genet (*Genetta genetta*), stoat (*Mustela erminea*), Iberian wolf (*Canis lupus signatus*) and brown bear (*Ursus arctos*).

### Sampling design

#### Spatial scales

Two spatial scales, ‘landscape’ and ‘habitat’ scales were considered. The landscape scale (∼10 km^2^) comprises a river basin with the main habitat types of the region (Fig. 1A, 1B). The habitat scale (∼1−2 km^2^) comprises a habitat type within each landscape (Fig. 1B). We performed a factorial sampling protocol in 3 landscapes ×3 habitat types within each, resulting in a total of 9 sampling sites. The three studied landscapes (called ‘Seceda’, ‘Parada’ and ‘Ferramulín’) differ in cover of dominant habitat types and fleshy-fruited plant assemblage (Fig. 1A, Table S1, Table S2). A clear gradient in the cover of the different dominant habitat types can be identified among the three landscapes, with Parada being the most forested landscape, followed by Ferramulín and finally Seceda (Table S1). The same pattern is observed for the cover of pastures and crops and the opposite is observed for the cover of scrubland (Table S1). The highest abundance of wild fleshy-fruited species was found in Ferramulín, followed by Parada and Seceda; whereas the lowest abundance for cultivated fleshy-fruited species was found in Parada (Table S2).

The three habitat types studied within each landscape were chestnut woodlands, scrublands and ‘mosaics’. Chestnut woods have little or no understory because of clearing, although some wild fleshy-fruited plants can be found in less managed areas or at woodland edges (Fig. 1C, Table S2). Scrublands are very poor in fruiting plant species and only bramble and some alder buckthorn or rowan can be found (Fig. 1C, Table S2). Finally, one of the richest habitats of fleshy-fruited species are mixtures of small patches (usually <5 ha.) of woodlands (abandoned chestnut woods or other woodland types), scrublands, pastures and crops, generating ‘mosaics’ in which cultivated and, particularly, wild fruits are abundant (Fig. 1C, Table S2). These habitats cover most of the territory, and have contrasting levels of vegetation structure, successional stage and degree of human disturbance, which determine the fleshy-fruited plant assemblage at a narrow scale (Fig. 1A, Table S1, Table S2). The highest abundance of wild fleshy-fruited species was found in mosaics, followed by chestnut woodlands and scrubland; whereas the highest abundance for cultivated fleshy-fruited species was found in mosaics and woodlands (Table S2).

#### Collection of faeces

We searched for faeces in one fixed-transect along existing walking paths (mean ± SD  = 1.78±0.56 km length) within each site (Table S2), with a total distance sampled around 16 km. We assumed that sampling effort was similar among sites. This assumption relied on three facts: (1) transect length did not differ significantly among landscapes or habitats (Kruskall-Wallis test, both *P*>0.210); (2) the number of faeces found per meter sampled was similar among landscapes and habitats (Kruskall-Wallis test, both *P*>0.288); and (3) the length of transect was not associated with the number of faeces found per meter sampled (Spearman's rank correlation analysis: r_s_ = 0.460, *P* = 0.212, n = 9).

To detect temporal variations of fruit consumption by carnivorous mammals (i.e. the seed deposition phenology), we performed monthly surveys (last week of each month) during the entire fruiting season from August 2007 to January 2008 (n_surveys_ = 6). Due to logistic constrains only five out of nine transects were surveyed in November (Table S2). The main criteria for the identification of faeces at the level of carnivore species were shape, size, colour, and smell in combination. This procedure is commonly used for the identification of carnivore faeces [8], [15], [17], [19], [21], [22]. Faeces that could not be properly assigned to any carnivore species were classified as ‘non-identified’, but also considered for subsequent analysis (see below).

Faecal samples were broken, cleaned, and seeds classified and identified to the species level whenever possible using reference collection (unpublished data). From each faecal sample collected, seeds were counted. All plant species dispersed by carnivores in our study area had <5% damaged seeds (unpublished data) [8].

#### Plant abundance

Abundance and composition of those fleshy-fruited species recorded in carnivore faeces were calculated by counting the number of adult plants within a 10-m belt on each side of the fixed transects (mean ± SD area sampled per transect was 3.6±1.1 ha.). All plants bearing more than 100 fruits were considered as adult plants [36]. Plant abundance was expressed as number of fruiting plants per hectare (Table S2). Bramble and bilberry (*Vaccinium myrtillus*) have a high vegetative spread and thus delineation of individual plants is often impossible. As a solution, we estimated visually the area covered by each ramet (length × width; m^2^) and calculated the cover of these two species in each transect as Cover (%)  = 100× (area covered by ramets/transect area) (see Table S2).

### Data analyses

Our purpose in this study was to examine frugivory and spatial patterns of seed deposition by carnivores as a disperser guild; therefore, we pooled samples of all carnivore species for subsequent analysis. In addition, since our goals were related to spatial patterns of seed deposition (not temporal patterns), we pooled all faeces for spatial analyses regardless the month that each sample was collected.

The frequency of occurrence (%) of seeds from fleshy-fruited species in faeces was calculated in order to estimate the importance of different fruit types in the diet of carnivores and the intensity of seed deposition. Spatial patterns of seed deposition were assessed at three different levels: (1) seed deposition frequency by carnivores, with all plant species pooled; (2) seed deposition frequency from different fruit types, i.e. wild and cultivated fruits; and (3) seed deposition frequency of different plant species. Because not all transects were surveyed in November, this month was excluded from the subsequent spatial analyses. Although 6 rose and 8 bramble species occur in the region (Javier Amigo, personal communication), we were unable to identify the seeds of each species. We therefore grouped these species as *Rosa* spp. and *Rubus* spp. for analysis purposes.

We categorized all faeces as a binary variable according to the presence/absence of a given seed type (all fleshy-fruited species together, wild and cultivated fruit types and individual fleshy-fruited species). Then, for each group of seeds, the effects of landscape, habitat, and their interaction (L × H) on seed deposition were tested using Generalized Linear Models (GLMs) on a factorial ANOVA-type design with binomial errors distribution and logit-link function. We considered different sample size for each analysis: first, to analyze spatial variation in seed deposition for all fleshy-fruit species, all faeces collected were used; second, for spatial variation in seed deposition of different fruit types, we only considered faeces containing seeds; and third, for individual plant species analyses, only faeces with seeds collected during the months in which the species analyzed occurred in faeces were used.

At the individual species level, GLMs were performed only for those plant species appearing in a minimum of 20 samples and with a frequency of seed occurrence in faeces >5%. Applying these criteria, the wild species analyzed were *Frangula alnus*, *Prunus spinosa*, *Rubus* spp. and *Sorbus aucuparia*, and the cultivated species were *Ficus carica* and *Prunus avium*. Due to the fact that *Malus domestica* and *Pyrus domestica* overlap in fruiting phenology producing similar fruits and usually occur in the same orchards, both species were analyzed together (hereafter ‘*Malus-Pyrus*’) because individually they did not satisfy the criteria for analysis. For simplicity, these seven species will hereafter be denoted through the text by the genus name, with the exception of the cherry tree and blackthorn which will be denoted as *P. avium* and *P. spinosa*, respectively.

Furthermore, for a given seed type, we evaluated the importance of landscape, habitat and their interaction on frequency of seed deposition. To do this, we took into account the total explained deviance by each GLM (the difference between the explained deviance in the null model, that is, the intercept-only model, and the residual deviance of the model) and we calculated the percentages of relative variance (RV) accounted for by each variance component (landscape, habitat, and interaction term) using the deviance quotients provided by GLMs [2].

Quantity components of seed deposition were calculated for every species using: (1) the abundance of faeces containing its seeds per km, and (2) the number of seeds per km of transect. Relationships between frequency of occurrence and between quantity of seed deposition (either in number of faeces or seeds per km), and plant abundance (density or cover) at the nine sampling sites were tested by the Spearman's rank correlation analyses.

All statistical analyses were performed using the “R” statistical software V.2.8.0 [37].

## Results

A total of 1077 carnivore faeces were collected (mean ± SD  = 178±70 per month; 203±41 when excluding November data). Among landscapes, 42% of faeces were collected in Parada, 33% in Ferramulín, and 25% in Seceda; whereas between habitats, 49% of faeces were collected in chestnut woodlands, 27% in mosaics and 24% in scrublands (Table S3). Out of the total number of faeces collected, 37% were of red fox, 35% of pine and stone martens (pooled together due to difficulties in identifying each species), 11% of badger and 1% of other species (genet, weasel, stoat, wolf or brown bear). We were unable to classify 16% of faeces because of their high level of degradation.

Out of 1077 faeces collected, 705 faeces (65%) contained seeds and more than 106000 seeds were recovered, with 78% being from wild species (Table 1, Table S3). We identified at least 14 fleshy-fruited species, 8 wild and 6 cultivated species (Table 1, Table S3).

**Table 1:** Descriptive statistics of the 14 fleshy-fruited species consumed by carnivorous mammals in O Courel Mountains (NW Spain) during the 2007–2008 fruiting season (August to January), all studied sites and months combined.

Fleshy-fruited species	Total seeds	Seeds per faecal sample		
	recovered	mean	range	*n*
Wild species				
* Crataegus monogyna*	12	2.4	1–4	5
* Frangula alnus*	3433	53.6	2–250	64
* Prunus spinosa*	300	9.7	1–27	31
* Pyrus cordata*	185	7.4	1–35	25
* Rosa* spp.	338	16.9	1–82	20
* Rubus* spp.	56284	203.0	9–1200	278
* Sorbus aucuparia*	3468	38.0	8–208	52
* Vaccinum myrtillus*	19225	1478.8	20–3870	13
Cultivated species				
* Ficus carica*	17192	129.6	1–940	133
* Malus domestica*	48	2.3	1–8	21
* Prunus avium*	5377	27.2	1–300	197
* Prunus domestica*	40	2.0	1–4	20
* Pyrus communis*	60	3.0	1–10	20
* Vitis vinifera*	56	9.3	7–13	6
All species combined	106018	151.6	1–3870	705

### Plant species involved and seasonality

In those faeces with seeds, wild and cultivated species appeared in similar proportions 58% and 53%, respectively (Z-test = 0.825, *P* = 0.410). The wild species with the highest frequency of occurrence were *Rubus*, *Frangula* and *Sorbus*, whereas seeds from *P. avium* and *Ficus* were the most frequent cultivated species (Table 1, Table S3). Simultaneous frugivory of both fruit types was observed in 13% of faeces with seeds (n = 85 faeces), where *Rubus* (80% of faeces) and *Ficus* (61%) were the most common wild and cultivated species, respectively. In 14.5% of the faeces we found seeds belonging to more than two plant species.

**Figure 2:**
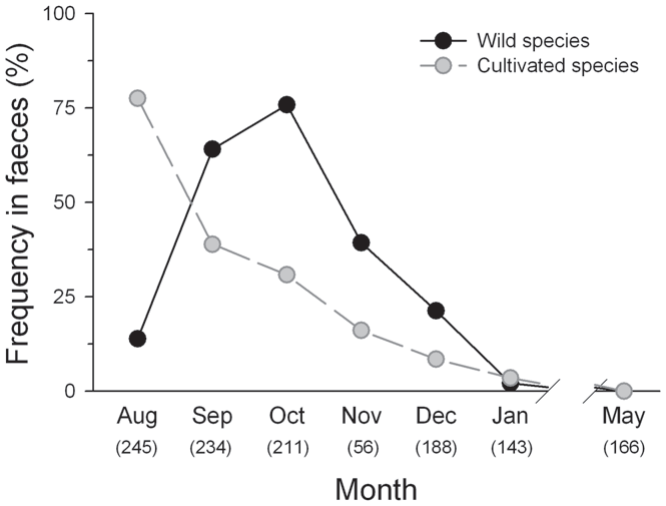
Temporal variation of fruit consumption (% seed occurrence in faeces) by carnivores of wild and cultivated fleshy-fruits. Percentages were calculated over the total number of faeces (i.e. the whole diet). Numbers below the months denote the number of faeces collected in each survey. Note that we included data from May 2008 (n _faeces_ = 166; out of this study) to show the whole annual variation of fruit consumption.

**Figure 3:**
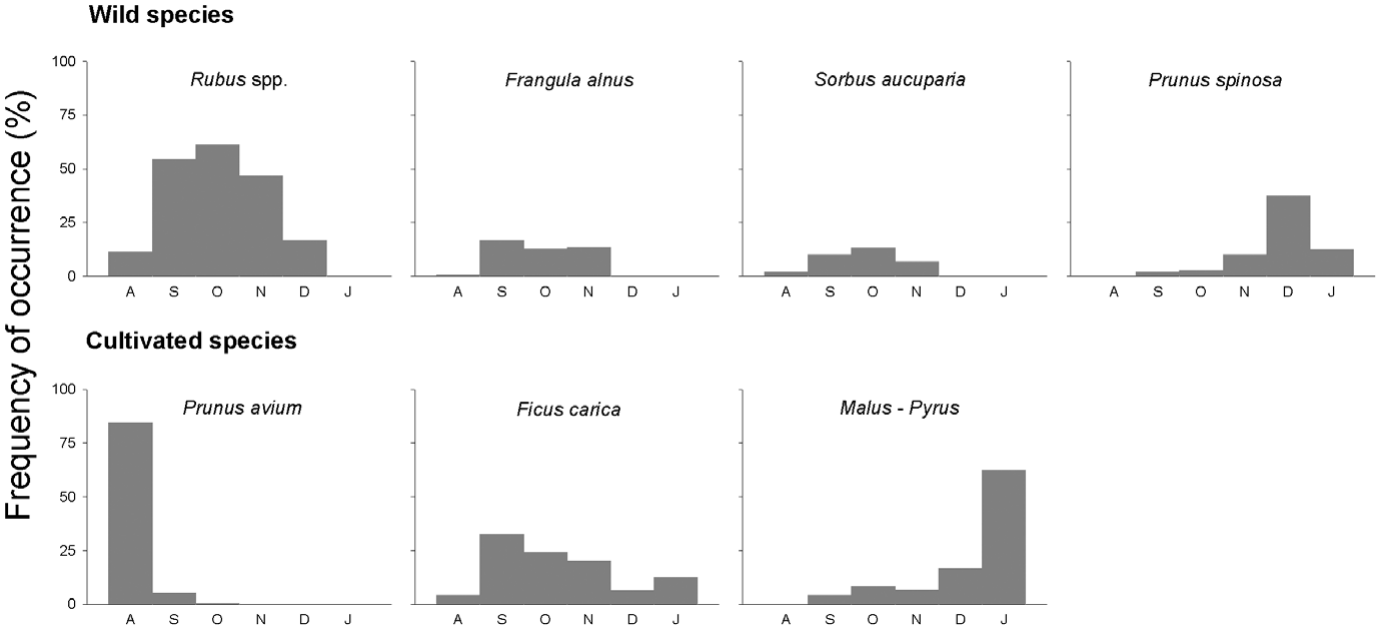
Temporal variation (August to January) of fruit consumption (% seed occurrence in faeces) by carnivores of different fleshy-fruited species. Note that contrary to figure 2 percentages were calculated over the number of faeces containing seeds (i.e. the frugivorous diet).

Overall, the frugivorous diet of carnivores showed a marked seasonality, with a peak (seeds in >90% of faeces) extending from late summer to autumn, and decreasing to a minimum (0–6%) in early winter and spring (Fig. 2). Seasonal patterns of frugivory were different between fruit types: whereas wild fruits were frequently consumed during autumn months, consumption of cultivated species showed a peak in the summer, which might be a result of the high consumption of *P. avium* cherries (Fig. 2 and 3). Also, individual fruit species showed a marked seasonality in frequency of occurrence in carnivore faeces. Among wild fruits, *Rubus*, *Frangula* and *Sorbus* represented most of the frugivorous diet during late summer-autumn (from September to October, Fig. 3), excepting for *P. spinosa* for which the peak of occurrence was in December (Fig. 3). On the other hand, cultivated fruits occurred either in summer (*P. avium*), autumn (*Ficus*) or winter (*Malus-Pyrus*; Fig. 3).

### Spatial patterns of seed deposition

#### Spatial variation in seed deposition: total and fleshy-fruited types

**Figure 4:**
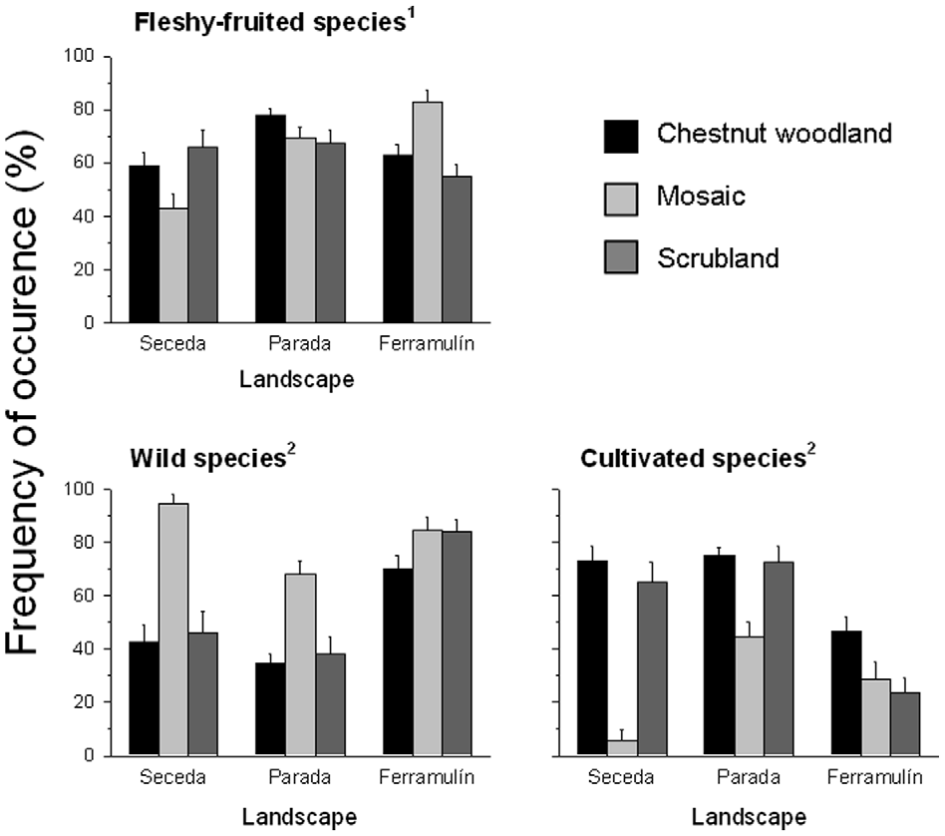
Patterns of seed deposition by carnivores at different spatial scales (landscapes and habitat types) showing the values (mean ± SE) for the frequency of seed deposition of fleshy-fruited species (all pooled), and wild and cultivated species. Superscripts denote the diet for which frequency of seed deposition was calculated; 1: whole diet, *n*  =  total faeces collected; 2: frugivorous diet, *n*  =  faeces containing seeds.

We detected a strong spatial heterogeneity of seed deposition by carnivores. When all species were analyzed together, the frequency of seed deposition varied significantly among landscapes (RV = 43%) and among habitats within landscapes, although in a different way within each (L × H; RV = 46%; Table 2, Fig. 4).

**Table 2:** Results of Generalized Linear Models (GLMs) analyzing the effect of landscape and habitat types on seed deposition by carnivorous mammals in O Courel Mountains (NW Spain) during the 2007–2008 fruiting season, considering separately the presence in faeces of seeds from all fleshy-fruited species, wild or cultivated species and different fleshy-fruited species.

		Source of variation								
Fruit item	*n*	Landscape			Habitat			L × H		
		χ^2^_2_	*P*	RV	χ^2^_2_	*P*	RV	χ^2^_4_	*P*	RV
All fleshy-fruited species	1021	23.2	***	43	5.6	ns	11	24.9	***	46
Wild species	675	62.6	***	60	53.6	***	26	12.6	*	14
* Frangula alnus*	619	51.2	***	61	22.5	***	25	12.0	*	14
* Prunus spinosa*	456	27.8	***	57	19.8	***	39	2.0	ns	4
* Rubus* spp.	667	20.8	***	58	9.6	**	27	5.4	ns	15
* Sorbus aucuparia*	619	73.0	***	58	22.2	***	18	29.4	***	24
Cultivated species	675	50.2	***	37	60.4	***	44	26.2	***	19
* Ficus carica*	675	9.0	*	28	19.8	***	61	3.7	ns	11
* Malus-Pyrus* §	456	1.1	ns	8	11.4	**	72	3.2	ns	20
* Prunus avium*	619	51.8	***	53	19.9	***	20	27.1	***	27

Relative variance (RV) explained by frequency of frugivory accounting for landscape, habitat and their interaction (L × H) derived from GLMs are also shown. *n*  =  number of faecal samples used for each variable analyzed (see text for details). Parameter estimates (*β*) ± SE in the models are given in Table S4.

§Seeds from *Malus domestica* and *Pyrus communis* were pooled for data analyses (see text for details).

*P<0.05;

**P<0.01;

***P<0.001; ns, no significant effects.

The analyses at the fruit type level showed that the frequency of seed deposition was significantly different among landscapes, habitats and among habitats within the same landscape (Table 2, Fig. 4). At the landscape scale, wild species were more frequently deposited in Ferramulín, whereas cultivated species were more frequently deposited in Parada (Fig. 4). At the habitat scale, wild species were more frequently deposited within the mosaics, whereas cultivated species were more deposited within chestnut woodlands and scrublands (Fig. 4). Despite this general pattern, such between-habitat differences in the frequency of deposition between both fruit types were landscape-dependent (Fig. 4), as shown by the significant effects of the interaction term (L × H; Table 2). Summarizing, whereas the most important source of spatial variation in seed deposition of wild species was the landscape scale (RV = 60%; Table 2), it was the habitat scale for cultivated species (RV = 44%; Table 2).

#### Spatial variation in seed deposition: individual species

**Figure 5:**
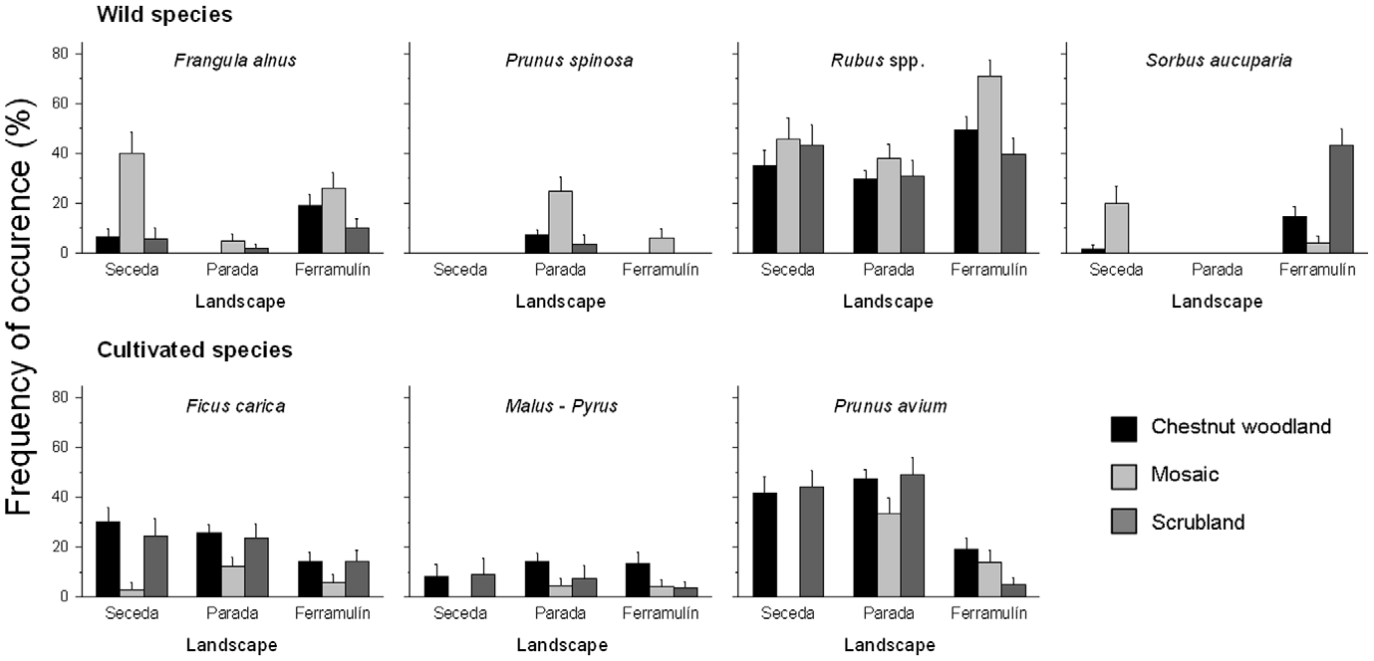
Patterns of seed deposition by carnivores at different spatial scales (landscapes and habitat types) showing the values (mean ± SE) for the frequency of seed deposition of different fleshy-fruited species.

High spatial heterogeneity in seed deposition was also found at the species level (Table 2, Fig. 5). Indeed, seed deposition differed significantly among landscapes and habitats for all wild fruit species (*Frangula*, *Rubus*, *P. spinosa* and *Sorbus*; Table 2, Fig. 5). Moreover, the interaction L × H showed a significant effect in two species (*Frangula* and *Sorbus*; Table 2, Fig. 5). *P. spinosa* was more frequently deposited in the most forested landscape (Parada), whereas *Frangula* and *Sorbus* were more deposited in the landscapes with the greater proportions of scrublands, pastures and crops (Seceda and Ferramulín; Fig. 5, see also Fig. 1). Although differing significantly among them, *Rubus* seeds were broadly deposited across all landscapes (Fig. 5). Three out of four wild species (*Frangula*, *Rubus* and *P. spinosa*) showed the highest rates of seed deposition within mosaic habitats (Fig. 5), whereas *Sorbus* seeds were more frequently deposited within scrublands (Fig. 5).

Among cultivated species, only deposition of *Ficus* and *P. avium* seeds differed among landscapes, with both species being more frequently deposited in the most forested landscape (Parada) where in addition, the lowest abundance of cultivated fruits occurred (Table S2). Seed deposition of cultivated species differed particularly among habitats and the three species considered showed a general pattern: the highest frequency of seed deposition occurred in chestnut woodlands and scrublands, while the lowest occurred in mosaics (Table 2, Fig. 5). The interaction L × H only showed significant effects on *P. avium*.

For the four wild species, landscape was the most important source of spatial variation in seed deposition (range 57–61%; Table 2). However, for the three cultivated species we did not find consistent patterns: most of the variance was accounted for by habitat for *Ficus* (RV = 61%) and *Malus-Pyrus* (RV = 72%) whereas the most important source of variation in *P. avium* was found at the landscape scale (RV = 53%, Table 2).

### Spatial concordance between seed deposition and plant abundance

We detected a general trend towards spatial concordance between the local abundance of fleshy-fruited plants and seed deposition at the three levels considered (i.e. frequency of occurrence, number of faeces and number of seeds per km) for all wild species (r_s_≥0.398; Table 3). However, such spatial concordance was only significant in *Frangula* and *P. spinosa* along these three levels (r_s_≥0.700, *P*≤0.036; Table 3). Conversely, we found a general lack of significant spatial concordance for cultivated species (r_s_ = from − 0.417 to 0.376, *P*≥0.265; Table 3).

**Table 3:** Values and significance level of the Spearman rank correlation coefficients between seed deposition by carnivorous mammals, both in relative (% of occurrence in faeces) and absolute (number of seeds and faeces km^−1^) terms, and the local abundance of different fleshy-fruited species (density or cover) at the 9 sampling sites**.**

	Seed dispersal estimates					
	Frequency of occurrence		Faeces km^−1^		Seeds km^−1^	
Fleshy-fruited species	*r*_s_	*P*	*r*_s_	*P*	*r*_s_	*P*
Wild species						
* Frangula alnus*	0.700	0.036	0.867	0.002	0.800	0.010
* Prunus spinosa*	0.800	0.010	0.800	0.010	0.700	0.036
* Rubus* spp.	0.567	0.111	0.500	0.170	0.400	0.286
* Sorbus aucuparia*	0.398	0.289	0.468	0.204	0.468	0.204
Cultivated species						
* Ficus carica*	0.287	0.454	0.248	0.521	0.376	0.318
* Malus-Pyrus* *	0.017	0.966	0.220	0.569	−0.136	0.728
* Prunus avium*	−0.417	0.265	−0.067	0.865	0.367	0.332

*Data from *Malus domestica* and *Pyrus communis* were pooled for data analyses (see text for details).

## Discussion

Carnivorous mammals fed on a considerable amount of fleshy-fruits in the O Courel Mountains, as has been reported in other studies in temperate regions [8], [15], [17], [19], [34], suggesting that fruits may be an important food resource for carnivores in this area. Fruit consumption showed a strong seasonality with a peak in autumn months, reflecting the seasonal patterns of fruit availability in temperate climate zones [38]. The large amount of seeds appearing in a significant proportion of faeces indicates that carnivores play an important role as dispersal agents even in disturbed areas [8], [25], although it is noteworthy that the number of fruit species consumed was quite similar for wild and cultivated plants.

Although morphological characters of fleshy-fruits eaten by carnivores exhibit a great variability, several authors have found that a high proportion of these fruits are large in size, low in seed-burden and have a notable odor [8], [10], [34], traits that define properly cultivated fruits. Our results suggest that a stronger preference for cultivated fruits by carnivores may occur in anthropogenic areas, regardless their abundance and distribution [15]. First, wild and cultivated seeds were found in carnivore faeces in similar proportions (58 vs. 53%, respectively) even when cultivated plants were much less abundant than wild plants. Second, despite 78% of seeds recovered were of wild fruits, estimations of the number of wild and cultivated fruits eaten by carnivores taking into account the average number of seeds per fruit showed that 53% of fruits were of cultivated fruiting plants (unpublished results). Third, the spatial patterns of seed deposition of cultivated species and the absence of spatial concordance between seed deposition and plant abundance found, suggest that carnivores actively sought cultivated fruits over wild fruits regardless of the habitat type [22]. Finally, this idea is also supported by the fact that the highest frequency in seed deposition of cultivated species was found in Parada (66% of faeces with seeds), even when this landscape showed the highest abundance of wild fruiting plants and the lowest abundance of cultivated fruiting plants.

### Spatial patterns in seed deposition by carnivorous mammals: Why does the fruit type matter?

Seed deposition by carnivorous mammals was spatially structured among landscapes and between habitats within landscapes. However, the strength of such variation and its relative importance were highly species-specific, with the fruit type being a determinant plant trait. Although seed deposition of wild species varied between both spatial scales, the highest degree of variation occurred among landscapes. Conversely, for two of the three cultivated species the habitat scale was the main source of spatial variation. Germination and establishment of *P. avium* in semi-natural habitats may explain why this species did not show the same spatial pattern than the rest of cultivated fleshy-fruited species.

The role of the landscape in generating such strong differences in seed deposition patterns of wild species may be explained by the high heterogeneity in presence and abundance of these species at this scale. Wild species varied vastly among landscapes due to existing differences in composition and abundance of species-specific favourable habitats, while cultivated species occur in small orchards within the three landscapes studied. Along these lines, although plant abundance was quite different among habitat patches within the same landscape, marked differences in fruit availability at broader scales (landscape) might constrain the spatial variation in plant-frugivore interactions at smaller scales (habitat) [23]. So, contrary to some wild species such as *P. spinosa* and *Sorbus*, the availability of cultivated species could be ensured at a landscape scale across the region, which may explain why the habitat scale was the most important source of variation of seed deposition for cultivated species.

Seed deposition was also spatially structured among habitat types within landscapes. Seeds of wild fruits tended to end up in mosaics, whereas those of cultivated fruits tended to be deposited in chestnut woodlands and scrublands. These distinct patterns between fruit types were also detected among individual species. Due to the high abundance of wild fruiting plants in the mosaics, the high frequency of seed deposition occurring within them is not a surprise. In fact, we found a general trend for a spatial concordance (all r_s_ positive although only significant for *Frangula* and *P. Spinosa*) between plant abundance and seed deposition of wild species, in both relative and absolute terms. An interesting result was the higher frequency of seed deposition of cultivated seeds in woodlands and scrublands. Fruit orchards are distributed together with chestnut woodlands around villages, however, cultivated fruiting plants are almost absent within scrublands. Consequently, we found a clear lack of spatial concordance between plant abundance and seed deposition for cultivated species.

We argue that these results might reveal two important aspects of the disperser assemblage studied. Firstly, home range sizes of the main carnivore species consuming fruits within the region (red fox, badger, pine and stone martens) can be larger than the habitat scale (sometimes home ranges above 10 km^2^) [39]–[42], allowing seed movement among habitat types within landscapes favouring dispersal from fruit-rich habitat patches to habitats poor in fleshy-fruited plants such as the scrublands [19]. Secondly, the intensity of seed deposition between habitat types can be highly influenced by fruiting plant features: while seeds of wild species were more frequently deposited at sites where adult fruiting plants were more abundant (self-reinforcing effect) [22], for cultivated species this did not occur. The main cultivated species in O Courel are groups of trees in orchards surrounding small villages (e.g. cherry trees, fig trees or apple trees). Despite that the number of individual cultivated plants may be small, their large fruit crops and the fact that their fruits typically fall to the ground after ripening make fruiting trees predictable food-rich patches for carnivores [8], [15], [43], [44]. Habitat use by carnivores can be influenced by fruit-rich patches [22], [41], and cultivated trees in orchards can be considered fruit-rich patches, at least in terms of fruit quality. As mentioned above, cultivated fruits had a lower seed burden than wild fruits, and for the same amount consumed they must provide a higher nutritive reward than wild fruits [15]. The feeding behaviour of frugivores influenced by the abundance of their “preferred” fruits (high-reward) may determine strong spatial differences in the patterns of frugivory at “non-preferred” (low-reward) fruit species [45].

We stress that other carnivore behaviours may play important roles in the seed deposition patterns observed. Contrary to birds, in which the spatial deposition of faeces is mainly associated with the location of perching sites [4], scent marking with faeces is a key behaviour in carnivores for territorial marking as well as inter- and intra-specific communication [46], [47]. In addition, in heterogeneous landscapes, distribution patterns and habitat use of carnivores varies among habitat patches [48]. Therefore, the spatial patterns of seed deposition reported in this study were likely influenced by multiple and complex carnivore behaviours such as habitat selection, foraging and territorial behaviours. Furthermore, possible differences in carnivore assemblages (diversity and abundance) among landscapes as a result of different habitat structure and composition may have also played a role.

Finally, since we sampled the same transects repeatedly within each site data may not be completely independent in terms of the number of individual carnivores that produced the faeces we collected. A possible solution to reduce this kind of pseudoreplication would be increasing the number of landscapes, which has several logistic constraints given the periodicity of our sampling. Another option would be increasing the number of transects per site or even the number of habitat patches per landscape, but this would not solve the problem either because home range sizes of the carnivore species studied here are typically larger than the mean patch size (∼1−2 km^2^). In fact, pseudoreplication involving the collection of several faecal samples that could have been produced by the same individual is probably the rule in studies on frugivory and seed dispersal by carnivores and other mammals [8], [14]–[17], [19]–[22], [24]–[27], [34] as well as by birds [2], [3], [26], [27], particularly, those based on the sampling of faeces/droppings within different spatial units (e.g. transects, plots, or seedfall trays).

### Beyond seed dispersal: implications for plant recruitment

Carnivore gut processes usually does not compromise seed viability. In general, negligible seed damage ratio and neutral or positive effects on seed germination have been documented [8], [14], [18]–[20]; therefore, we could roughly consider seed deposition and seed dispersal as similar terms. Along these lines, the end of the dispersal phase means the beginning of post-dispersal processes associated with plant recruitment (seed survival, germination and seedling establishment). Plant recruitment is a multiphase process and post-dispersal stages may override the differences among habitats in carnivore-mediated seed deposition [5]. In the case of anthropogenic systems, the success or failure of plant recruitment can be strongly induced by human-management practices [49]. For example, we found high levels of seed deposition in chestnut woodlands but seedling establishment is unlikely due to understory clearings for the harvesting of sweet chestnuts; therefore, seedling establishment may be possible only at woodland edges. However, as in many other rural areas of Europe, O Courel is undergoing a swift process of depopulation and land abandonment, which began during the past five decades and it is still occurring [32], [50]. As a result, the region is undergoing a marked change in landscape structure [32]. We found that carnivores deposited a considerable quantity of seeds in mosaics and scrublands, two habitat types that are very susceptible to short-term changes in vegetation composition [32]. The lack of current human-management in the region offers a good opportunity for seedling establishment in scrublands and abandoned patches in mosaics. Thus, carnivores might be playing an important role as ‘restorers’ and ‘habitat shapers’ under the current scenario [11]. This role must be especially relevant in fruit-poor habitat types usually avoided by the avian frugivore assemblage as the scrublands.

### Conclusions

To our knowledge, this study provides a novel approach for evaluating spatial patterns of seed deposition by carnivorous mammals considering the relative contribution of different spatial scales. Our research demonstrates that seed deposition by carnivores is a complex and scale-dependent process which seems to be modulated, among other factors, by the assemblages of fleshy-fruited plants and the spatial behaviour of dispersers under the influence of fruit features (e.g. wild or cultivated). Thus, we encourage the use of multiple replicates at different spatial scales to study properly the spatial patterns of seed deposition by large-sized frugivores and associated ecological processes.

Our results support the inter-fruit type competition hypothesis in anthropogenic areas [15], which state that the preference of cultivated fruits by carnivores can result in a reduction of their dispersal services to wild species. The fact that the fruiting peaks of wild and cultivated species were non-overlapping must minimize the interference of cultivated plants on carnivore-wild species mutualism in our study area. However, we could expect a stronger interference of non-native plants (either cultivated or alien species) in those cases in which fruiting peaks were highly overlapped.

Finally, the role of carnivorous mammals as seed dispersers (with large home ranges and longer gut retention time with respect to birds), seems to be important not only for gene flow between isolated plant populations [26], but also for colonization and reforestation of new vacant habitats after their abandonment [25].

## Supporting Information

Table S1 Cover percentage of dominant habitat types at landscape scale (∼10 km2) in the three studied landscapes in O Courel Mountains (NW Spain). We standardized the area considered within each landscape by buffering all transects within each landscape with a buffer area equal to 10 km2 and merging these three buffers per landscape. Then, on the resulting surface, we obtained cover percentages (%) from A. Larrinaga, I. Pulgar and M. Maceira, unpublished digital habitat map using ArcGIS 9 (Esri Inc., Redlands, CA, USA).
(0.03 MB DOC)

Table S2 Characteristics of the sampling transects in the nine studied sites in O Courel Mountains (NW Spain) (three landscapes and three habitat types within each). Plant abundance is expressed as plants ha−1, except for Rubus spp. and Vaccinium myrtillus (*) for which is expressed as covers (%) along the sampling transects. (See Methods for details on plant abundance estimation).
(0.06 MB DOC)

Table S3 Number of seeds recovered of the 14 fleshy-fruited species consumed by carnivorous mammals in O Courel Mountains (NW Spain) during the 2007-2008 fruiting season (August to January) for each of the nine sampling transects. Numbers between brackets denote the number of faeces collected in each transect.
(0.05 MB DOC)

Table S4 Parameter estimates (β) ± SE in the Generalized Linear Models analyzing the effect of landscape and habitat types on seed deposition by carnivorous mammals in O Courel Mountains (NW Spain) during the 2007-2008 fruiting season.
(0.04 MB DOC)
