# Supplementary material for: Frugivory and Spatial Patterns of Seed Deposition by Carnivorous Mammals in Anthropogenic Landscapes: A Multi-Scale Approach
Source: PLoS One. 2011 Jan 21;6(1):e14569. doi: 10.1371/journal.pone.0014569 (PMC3024974; doi:10.1371/journal.pone.0014569)
Supplement: Table S1 — Cover percentage of dominant habitat types at landscape scale (∼10 km2) in the three studied landscapes in O Courel Mountains (NW Spain). We standardized the area considered within each landscape by buffering all transects within each landscape with a buffer area equal to 10 km2 and merging these three buffers per landscape. Then, on the resulting surface, we obtained cover percentages (%) from A. Larrinaga, I. Pulgar and M. Maceira, unpublished digital habitat map using ArcGIS 9 (Esri Inc., Redlands, CA, USA). (0.03 MB DOC) [file pone.0014569.s001.doc]

**Table S1**. **Cover percentage of dominant habitat types at landscape scale (~10 km^2^) in the three studied landscapes in O Courel Mountains (NW Spain).** We standardized the area considered within each landscape by buffering all transects within each landscape with a buffer area equal to 10 km^2^ and merging these three buffers per landscape. Then, on the resulting surface, we obtained cover percentages (%) from A. Larrinaga, I. Pulgar and M. Maceira, unpublished digital habitat map using ArcGIS 9 (Esri Inc., Redlands, CA, USA).

| Habitat type | SECEDA | PARADA | FERRAMULÍN |
| --- | --- | --- | --- |
| Mature deciduous woodland | 5.7 | 14.7 | 6.9 |
| Other woodland types | 7.6 | 23.9 | 16.7 |
| Scrubland | 84.5 | 44.4 | 64.7 |
| Pastures and crops | 1.8 | 13.6 | 9.7 |
| Others | 0.3 | 3.3 | 2 |
