# Supplementary material for: Frugivory and Spatial Patterns of Seed Deposition by Carnivorous Mammals in Anthropogenic Landscapes: A Multi-Scale Approach
Source: PLoS One. 2011 Jan 21;6(1):e14569. doi: 10.1371/journal.pone.0014569 (PMC3024974; doi:10.1371/journal.pone.0014569)
Supplement: Table S2 — Characteristics of the sampling transects in the nine studied sites in O Courel Mountains (NW Spain) (three landscapes and three habitat types within each). Plant abundance is expressed as plants ha−1, except for Rubus spp. and Vaccinium myrtillus (*) for which is expressed as covers (%) along the sampling transects. (See Methods for details on plant abundance estimation). (0.06 MB DOC) [file pone.0014569.s002.doc]

**Table S2. Characteristics of the sampling transects in the nine studied sites in O Courel Mountains (NW Spain) (three landscapes and three habitat types within each).** Plant abundance is expressed as plants ha^−1^, except for *Rubus* spp. and *Vaccinium myrtillus* (*) for which is expressed as covers (%) along the sampling transects. (see Methods for details on plant abundance estimation).

|  | SECEDA | | | |  | PARADA | | | |  | FERRAMULÍN | | | |
| --- | --- | --- | --- | --- | --- | --- | --- | --- | --- | --- | --- | --- | --- | --- |
| **Sampling transects** | Woodland | Mosaic | Scrubland | Total/mean |  | Woodland | Mosaic | Scrubland | Total/mean |  | Woodland | Mosaic | Scrubland | Total/mean |
|  |  |  |  |  |  |  |  |  |  |  |  |  |  |  |
| Transect length (m) | 1095 | 2215 | 1910 | 5220 |  | 2460 | 2130 | 2035 | 6625 |  | 2100 | 1060 | 1010 | 4170 |
| Altitudinal range (m. a.s.l.) | 760−800 | 500−640 | 750−990 | 500-990 |  | 500−640 | 590−630 | 770−1070 | 500-1070 |  | 840−950 | 920−970 | 870−970 | 840-970 |
| Number of surveys | 6 | 6 | 5 | 17 |  | 5 | 5 | 5 | 15 |  | 6 | 6 | 6 | 18 |
| Wild species abundance |  |  |  |  |  |  |  |  |  |  |  |  |  |  |
| *Crataegus monogyna* (Rosaceae) | - | 0.4 | 1.0 | 0.5 |  | 13.2 | 65.5 | 0.5 | 26.4 |  | - | 1.4 | 0.5 | 0.6 |
| *Frangula alnus* (Rhamnaceae) | 22.8 | 8.3 | 1.8 | 11.0 |  | - | 16.4 | 0.2 | 5.5 |  | 28.8 | 94.3 | 44.5 | 55.9 |
| *Prunus spinosa* (Rosaceae) | - | - | - | - |  | 9.5 | 28.6 | - | 12.7 |  | - | - | - | - |
| *Pyrus cordata* (Rosaceae) | - | - | - | - |  | 0.8 | 1.4 | 1.0 | 1.1 |  | - | - | 1.0 | 0.3 |
| *Rosa* spp. (Rosaceae) | - | - | - | - |  | 7.5 | 22.3 | - | 9.9 |  | 1.4 | 5.7 | - | 3.6 |
| *Rubus* spp. (Rosaceae)* | 1.6 | 1.7 | 14.1 | 5.8 |  | 8.3 | 21.3 | 1.5 | 10.4 |  | 14.6 | 27.7 | 4.4 | 15.6 |
| *Sorbus aucuparia* (Rosaceae) | - | 0.2 | - | 0.1 |  | 2.4 | 0.7 | 0.2 | 1.1 |  | 2.6 | 10.4 | 7.9 | 7.0 |
| *Vaccinum myrtillus* (Ericaceae)* | - | - | - | - |  | 1.8 | - | - | 0.6 |  | 1.6 | - | 0.3 | 0.6 |
| Cultivated species abundance |  |  |  | - |  |  |  |  |  |  |  |  |  |  |
| *Ficus carica* (Moraceae) | 2.3 | 0.7 | - | 1.0 |  | 0.4 | - | - | 0.1 |  | - | - | - | - |
| *Malus domestica* (Rosaceae) | 2.7 | 2.3 | - | 1.7 |  | 1.0 | - | - | 0.3 |  | 2.1 | - | - | 0.7 |
| *Prunus avium* (Rosaceae) | 3.6 | 0.9 | 0.3 | 1.6 |  | 3.2 | 2.6 | - | 1.9 |  | 2.4 | 11.8 | 4.5 | 6.2 |
| *Prunus domestica* (Rosaceae) | 0.5 | - | - | 0.2 |  | 0.6 | 0.2 | - | 0.3 |  | - | - | - | - |
| *Pyrus communis* (Rosaceae) | 1.4 | 2.3 | - | 1.2 |  | 1.0 | 0.2 | - | 0.4 |  | 0.5 | 0.5 | - | 0.3 |
| *Vitis vinifera* (Vitaceae) | - | 0.2 | - | 0.1 |  | - | - | - | - |  | - | - | - | - |
