# Supplementary material for: Frugivory and Spatial Patterns of Seed Deposition by Carnivorous Mammals in Anthropogenic Landscapes: A Multi-Scale Approach
Source: PLoS One. 2011 Jan 21;6(1):e14569. doi: 10.1371/journal.pone.0014569 (PMC3024974; doi:10.1371/journal.pone.0014569)
Supplement: Table S3 — Number of seeds recovered of the 14 fleshy-fruited species consumed by carnivorous mammals in O Courel Mountains (NW Spain) during the 2007-2008 fruiting season (August to January) for each of the nine sampling transects. Numbers between brackets denote the number of faeces collected in each transect. (0.05 MB DOC) [file pone.0014569.s003.doc]

**Table S3. Number of seeds recovered of the 14 fleshy-fruited species consumed by carnivorous mammals in O Courel Mountains (NW Spain) during the 2007–2008 fruiting season (August to January) for each of the nine sampling transects.** Numbers between brackets denote the number of faeces collected in each transect.

|  | SECEDA | | |  | PARADA | | |  | FERRAMULÍN | | |
| --- | --- | --- | --- | --- | --- | --- | --- | --- | --- | --- | --- |
| Fleshy-fruited species | Woodland | Mosaic | Scrubland |  | Woodland | Mosaic | Scrubland |  | Woodland | Mosaic | Scrubland |
| Wild species |  |  |  |  |  |  |  |  |  |  |  |
| *Crataegus monogyna* | - | - | - |  | 6 (2) | 6 (3) | - |  | - | - | - |
| *Frangula alnus* | 297 (4) | 980 (14) | 30 (2) |  | - | 169 (3) | 64 (1) |  | 434 (18) | 1054 (15) | 405 (7) |
| *Prunus spinosa* | - | - | - |  | 73 (8) | 137 (16) | 9 (1) |  | - | 81 (6) | - |
| *Pyrus cordata* | - | 45 (2) | - |  | - | 106 (8) | - |  | 23 (9) | 1 (1) | 10 (5) |
| *Rosa* spp. | - | - | - |  | 177 (10) | 114 (5) | - |  | 8 (1) | 35 (3) | 4 (1) |
| *Rubus* spp. | 5292 (25) | 4172 (17) | 3300 (16) |  | 8080 (56) | 6310 (30) | 2135 (17) |  | 9261 (48) | 13715 (42) | 4019 (27) |
| *Sorbus aucuparia* | 48 (1) | 376 (7) | - |  | - | - | - |  | 940 (13) | 176 (2) | 1928 (29) |
| *Vaccinum myrtillus* | - | 3645 (2) | - |  | 20 (1) | - | 350 (3) |  | 8640 (4) | - | 6570 (3) |
| Cultivated species |  |  |  |  |  |  |  |  |  |  |  |
| *Ficus carica* | 4689 (20) | 1125 (4) | 730 (9) |  | 5315 (50) | 1790 (10) | 1500 (13) |  | 1052 (15) | 146 (3) | 845 (9) |
| *Malus domestica* | 6 (4) | 2 (1) | - |  | 26 (7) | 5 (3) | - |  | 6 (4) | - | 3 (2) |
| *Prunus avium* | 783 (25) | - | 301 (15) |  | 2463 (84) | 423 (20) | 486 (26) |  | 503 (17) | 319 (7) | 99 (3) |
| *Prunus domestica* | 15 (6) | - | - |  | 1 (1) | - | 1 (1) |  | 19 (10) | 4 (2) | - |
| *Pyrus communis* | 1 (1) | - | 3 (2) |  | 27 (8) | - | 7 (2) |  | 4 (4) | 12 (2) | 6 (1) |
| *Vitis vinifera* | - | 10 (1) | - |  | - | 30 (3) | - |  | - | 16 (2) | - |
| All species combined | 11131 (86) | 10355 (48) | 4364 (44) |  | 16188 (226) | 9090 (101) | 4552 (64) |  | 20890 (143) | 15559 (85) | 13889 (87) |
