# Supplementary material for: Frugivory and Spatial Patterns of Seed Deposition by Carnivorous Mammals in Anthropogenic Landscapes: A Multi-Scale Approach
Source: PLoS One. 2011 Jan 21;6(1):e14569. doi: 10.1371/journal.pone.0014569 (PMC3024974; doi:10.1371/journal.pone.0014569)
Supplement: Table S4 — Parameter estimates (β) ± SE in the Generalized Linear Models analyzing the effect of landscape and habitat types on seed deposition by carnivorous mammals in O Courel Mountains (NW Spain) during the 2007-2008 fruiting season. (0.04 MB DOC) [file pone.0014569.s004.doc]

**Table S4.** **Parameter estimates (*β*) ± SE in the Generalized Linear Models analyzing the effect of landscape and habitat types on seed deposition by carnivorous mammals in O Courel Mountains (NW Spain) during the 2007–2008 fruiting season.** The levels not showed are included in the intercept.

|  | Parameter estimate (*β*) ± SE | | | | | | | | |
| --- | --- | --- | --- | --- | --- | --- | --- | --- | --- |
| Fruit item | Intercept | Scrubland | Mosaic | Parada | Seceda | Scrubland x Parada | Mosaic x Parada | Scrubland x Seceda | Mosaic x Seceda |
| All fleshy-fruited species | 0.5±0.2** | -0.3±0.2 | 1.1±0.3** | 0.7±0.2** | -0.2±0.3 | -0.2±0.4 | -1.5±0.4** | 0.6±0.4 | -1.7±0.5*** |
|  |  |  |  |  |  |  |  |  |  |
| Wild species | 0.8±0.2*** | 0.8±0.4* | 0.8±0.4 | -1.5±0.3*** | -1.1±0.3*** | -0.7±0.5 | 0.5±0.5 | -0.7±0.6 | 2.2±0.9* |
| *Frangula alnus* | -1.4±0.3*** | -0.8±0.5 | 0.4±0.4 | -18.1±0 | -1.1±0.6* | 16.4±0.0 | 16.2±0.0 | 0.6±1.1 | 1.8±0.7 |
| *Prunus spinosa* | -20.6±0.0 | -7e-3±0.0 | 17.9±0.0 | 17.9±0 | -7e-3±0 | -0.7±0.1 | -16.3±0 | 7e-3±0.0 | -17.8±0.0 |
| *Rubus* spp. | -0.1±0.2 | -0.4±0.3 | -0.9±0.4* | -0.8±0.3** | -0.6±0.4 | 0.4±0.5 | -0.5±0.5 | 0.7±0.5 | -0.5±0.1 |
| *Sorbus aucuparia* | -1.7±0.3*** | 1.5±0.4*** | -1.4±0.8 | -18.8±0.0 | -2.3±1.1* | -1.5±0.0 | 1.4±0.0 | -18.1±0.0 | 4.1±1.4** |
|  |  |  |  |  |  |  |  |  |  |
| Cultivated species | -0.1±0.2 | -1.1±0.4** | -0.8±0.4* | 1.2±0.3*** | 1.1±0.3** | 0.9±0.5 | -0.5±0.4 | 0.6±0.6 | -3.1±0.8*** |
| *Ficus carica* | -1.8±0.3*** | -0.1±0.5 | -1.1±0.7 | 0.7±0.3* | 0.9±0.4* | -0.1±0.6 | 0.1±0.7 | -0.3±0.7 | -1.7±1.2 |
| *Malus-Pyrus* | -1.9±0.4*** | -1.4±0.8 | -1.3±0.9 | 0.1±0.5 | -0.5±0.7 | 0.7±1.1 | 0.1±1.1 | 1.5±1.5 | -13.9±0.0 |
| *Prunus avium* | -1.4±0.3*** | -1.5±0.6* | -0.4±0.5 | 1.3±0.3*** | 1.1±0.4** | 1.6±0.7* | -0.2±0.6 | 1.6±0.8* | -16.8±0.0 |

**P* < 0.05; ***P* < 0.01; ****P* < 0.001
